# Spices as Sustainable Food Preservatives: A Comprehensive Review of Their Antimicrobial Potential

**DOI:** 10.3390/ph16101451

**Published:** 2023-10-12

**Authors:** Abdel Moneim E. Sulieman, Emad M. Abdallah, Naimah Asid Alanazi, Abdelaziz Ed-Dra, Arshad Jamal, Hajo Idriss, Abdullah Sulaiman Alshammari, Sohair A. M. Shommo

**Affiliations:** 1 Department of Biology, College of Science, University of Ha’il, Ha’il 55473, Saudi Arabia; n-almansor@hotmail.com (N.A.A.); arshadjamalus@yahoo.com (A.J.); 2 Department of Science Laboratories, College of Science and Arts, Qassim University, Ar Rass 51921, Saudi Arabia; 3 Laboratory of Engineering and Applied Technologies, Higher School of Technology, M’ghila Campus, Sultan Moulay Slimane University, Beni Mellal 23000, Morocco; abdelaziz_iaa@yahoo.fr; 4 Department of Physics, College of Science, Imam Mohammad Ibn Saud Islamic University (IMSIU), Riyadh 11623, Saudi Arabia; hiidriss@imamu.edu.sa; 5 National Center for Vegetation Cover Development and Combating Desertification, Riyadh 11195, Saudi Arabia; zssssty@gmail.com; 6 College of Education, University of Ha’il, Ha’il 55473, Saudi Arabia; susu.shommo@gmail.com

**Keywords:** food safety, preservatives, microbial inhibition, food-spoilage microorganisms, antibacterial agents, food industry, bio-preservation strategies

## Abstract

Throughout history, spices have been employed for their pharmaceutical attributes and as a culinary enhancement. The food industry widely employs artificial preservatives to retard the deterioration induced by microbial proliferation, enzymatic processes, and oxidative reactions. Nevertheless, the utilization of these synthetic preservatives in food products has given rise to significant apprehension among consumers, primarily stemming from the potential health risks that they pose. These risks encompass a spectrum of adverse effects, including but not limited to gastrointestinal disorders, the disruption of gut microbiota, allergic reactions, respiratory complications, and concerns regarding their carcinogenic properties. Consequently, consumers are displaying an increasing reluctance to purchase preserved food items that contain such additives. Spices, known for their antimicrobial value, are investigated for their potential as food preservatives. The review assesses 25 spice types for their inherent antimicrobial properties and their applicability in inhibiting various foodborne microorganisms and suggests further future investigations regarding their use as possible natural food preservatives that could offer safer, more sustainable methods for extending shelf life. Future research should delve deeper into the use of natural antimicrobials, such as spices, to not only replace synthetic preservatives but also optimize their application in food safety and shelf-life extension. Moreover, there is a need for continuous innovation in encapsulation technologies for antimicrobial agents. Developing cost-effective and efficient methods, along with scaling up production processes, will be crucial to competing with traditional antimicrobial options in terms of both efficacy and affordability.

## 1. Introduction

As per the population projections made by the United Nations, it is anticipated that the global human population will reach 9.7 billion by the year 2050. This situation is expected to induce a rise in worldwide food consumption [1]. Food security is currently a significant issue because of the growing globalization of the food industry, the need to produce the higher-quality foods demanded by consumers, market trends, and constantly changing legislation [2].

Food spoilage represents a metabolic progression leading to the deterioration of food items. It arises from a diverse array of processes, encompassing physical and chemical transformations as well as enzymatic and microbial actions, rendering them unsuitable or unpalatable for human consumption due to alterations in their sensory attributes. Some spoiled foods may not pose a health risk as they may lack pathogens or toxins, but their unappealing changes render them unattractive for consumption. [3]. The contamination and proliferation of food spoilage microorganisms have emerged as significant factors contributing to contemporary food loss [4]. In fact, the main causes of food spoilage are intrinsic food characteristics (such as enzymes, substrates, and oxygen), as well as the contamination that occurs during harvesting, slaughter, and processing, along with improper temperature management. The two main ways in which fresh foods can lose their quality are: (i) due to bacterial metabolism and growth, which can alter pH levels and lead to the formation of toxic compounds, gases, off-odors, and slime; (ii) the oxidation of pigments and lipids in foods that are high in fat, which can cause discoloration, unwanted flavors, and the formation of compounds with harmful biological effects [5].

Currently, food preservation represents a critical and significant concern, as escalating food prices have significantly impacted numerous developing regions since 2008 and continued thereafter, with many countries experiencing persistently elevated local food prices compared to historical levels [6]. Urgently, we require sustainable food processing technologies to address global food safety challenges. However, implementing new methods can introduce risks due to changes in microorganisms, production, the environment, and global trade. Careful assessment is necessary for safe and high-quality food products [7]. Artificial preservatives play a crucial role in extending the shelf life and preserving the quality of food for prolonged periods. However, there have been reports highlighting the potential side effects associated with the use of artificial preservatives. Adverse reactions to these preservatives can range from mild to life-threatening. Therefore, whenever feasible, it is advisable to opt for a diet that is free from preservatives [8]. In a previous study, while consumers possessed an understanding of some advantages that additives could offer, there persisted a prevalent preconception that additives were inherently undesirable. Consequently, these consumers harbored the belief that the presence of additives in our food products should be minimized [9]. In another pilot study, it was observed that 76.3% of the surveyed consumers expressed disagreement with the utilization of additives, with 83.6% indicating a perception of additives as potentially hazardous to health, particularly in the context of preservatives and colorings [10]. On the other hand, the adoption of natural antimicrobial compounds in food has garnered considerable interest in response to the escalating prevalence of food spoilage microorganisms and their heightened tolerance of conventional food preservation and processing techniques. Spices and herbs have garnered increasing significance in recent times as promising reservoirs of natural food preservatives. This stems from the escalating interest in exploring safe and efficient methods of natural food preservation [11]. The aim of this comprehensive review study is to evaluate and analyze recent scientific literature on the antimicrobial potential of spices, in the context of sustainable food preservation, as a promising natural alternative to artificial food preservatives that present numerous side effects on human health. 

## 2. Methodology

### 2.1. Flora Nomenclature

All recorded plant names underwent a meticulous verification process to ensure precision and conformity with established references, including the WFO Plant List (https://wfoplantlist.org/plant-list (accessed on 1 August 2023)), the International Plant Names Index (https://www.ipni.org/ (accessed on 1 August 2023)), the Plants of the World Online (https://powo.science.kew.org/ (accessed on 1 August 2023)), and the World Flora Online (https://www.worldfloraonline.org/ (accessed on 3 October 2023)). Botanical names and families are duly documented in Table 1 and in the main text of the study.

### 2.2. Antimicrobial Activity of Spices

**Figure 1:**
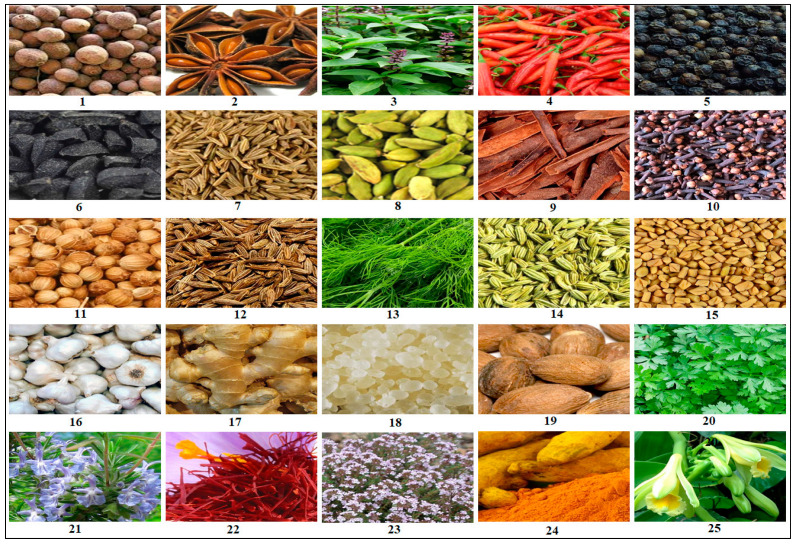
The 25 selected antimicrobial spices recommended as natural food preservatives (the numbers relate to the names of the spices, listed in the same order in Table 1).

Initially, a thorough and extensive exploration of the existing literature concerning the utilization of naturally occurring antimicrobial compounds for the purpose of food preservation was diligently undertaken. This investigative endeavor involved a meticulous online literature review, encompassing esteemed databases such as MDPI, Sage, Web of Knowledge, PubMed, Science Direct, Scopus, Springer, and Google Scholar. The search strategy employed a combination of focused keywords, including “natural antimicrobial agents”, “food spoilage microorganisms”, “spices and herbs”, “antibacterial”, and “anti-fungal”. The selected scope of the review encompassed studies and research articles related to natural antimicrobial compounds and their activities that were available from 2013 until 2023, exclusively considering content that was presented in the English language. The present review regards the following subjects as pertinent within the scope of the inquiry: spice-derived compounds, antimicrobial properties, innovative food preservation, food safety, food quality, the effectiveness of spice-derived compounds, chemical analysis, and emerging trends. The primary objective of this investigation was to thoroughly assess the phytochemical composition and elemental makeup inherent to spices and herbs. To achieve this, a comprehensive analysis of more than 120 pertinent studies was meticulously conducted, concentrating specifically on the identification and categorization of the bacterial and fungal species present in various food contexts. A detailed compilation of the collected research data is presented in Table 1, facilitating a comprehensive understanding of the synthesized information. Furthermore, the visual representation in Figure 1 provides an illustrative depiction of the 25 select spices that were judiciously chosen and critically examined as part of this systematic and thorough inquiry.

### 2.3. Inclusion and Exclusion Criteria

The defined inclusion criteria encompass spices and seasonings, including extracts, essential oils, or fractions originating from the roots, bulbs, trunks, leaves, seeds, flowers, and fruits, which exhibit antimicrobial potential and are in global usage as culinary and food additives, either alone or in combinations with other edible plants. The selection criteria for particular spice species encompassed multifaceted considerations, including their antimicrobial potential, widespread availability, cultural and culinary significance, safety profile, geographical provenance, extractability characteristics, and the extent of prior scholarly investigation.

The exclusion criteria include spices derived from non-angiosperm sources, such as ferns, lichens, algae, and seaweeds, which were excluded from the current investigation. In addition, those studies that were not published in the English language were systematically excluded from the scope of this research. The primary focus of this study centers on the in vitro evaluation of antimicrobial activity. Consequently, in vivo and in silico investigations were excluded from the purview of this review. Additionally, articles that examined the antimicrobial activity of spice mixtures or combinations, as well as the synergistic effects of spices with other compounds, were excluded from consideration, although they were implicitly referred to in the comments.

## 3. Results and Discussion

### 3.1. Artificial Food Preservatives

Food preservatives are compounds that are added to food with the purpose of preventing and retarding microbial spoilage. They inhibit, retard, or arrest processes such as fermentation, acidification, or other forms of decomposition in food. The primary goal of preservatives is to hinder the growth of microorganisms that could otherwise lead to food spoilage and potentially cause food poisoning. Additionally, they play a crucial role in extending the shelf life of products, allowing them to be distributed and sold to consumers while offering a longer shelf life [12].

Artificial food preservatives can be classified into different categories, based on their chemical composition and mechanism of action. Some common types include:

(i) Organic acids, such as ascorbic acid, acetic acid, and benzoic acid, are widely used in acidic food products to prevent microbial growth [13];
(ii) Nitrites and nitrates are commonly applied in cured meat products to inhibit the growth of *Clostridium botulinum* and provide color stabilization [13];
(iii) Sulfites are utilized in dried fruits, wines, and juices to control microbial activity and prevent browning [14];
(iv) Antioxidants, including butylated hydroxyanisole (BHA) and butylated hydroxytoluene (BHT), are used to extend the shelf life of products containing fats and oils [15];
(v) Antibiotics: nisin and natamycin are examples of antibiotics used to preserve a variety of food items [16].

**Figure 2:**
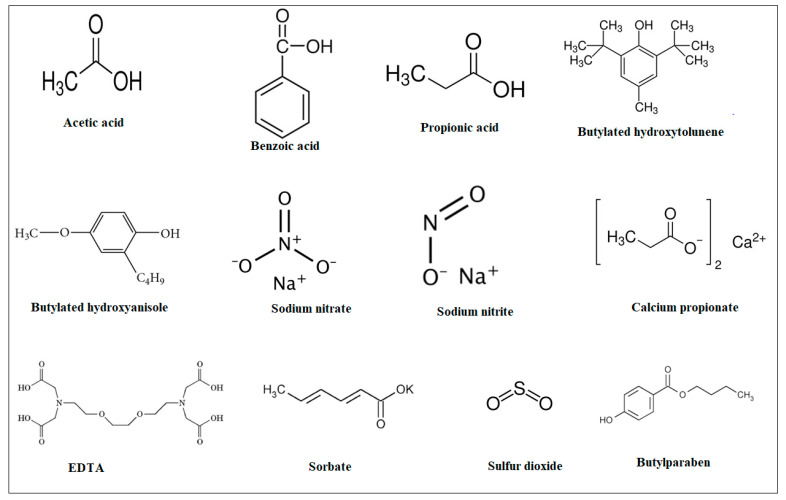
Example of some artificial antimicrobial food preservatives used in the food industry.

Presently, the United States Food and Drug Administration (US FDA) has approved over 3000 food additives. A comprehensive and authoritative list of all the diverse food additives utilized in the United States can be accessed at EAFUS (everything added to food in the United States) [17]. Food additives that possess antimicrobial properties, effectively safeguarding foods against the growth of microorganisms such as fungi and bacteria, are crucial in extending the shelf life of various food items. One of the oldest and most widely used preservatives is sodium chloride, commonly known as salt. Additionally, organic acids, including benzoic acid (E210), acetic acid (E260), sorbic acid (E200), propanoic acid (E280), and many more, are extensively utilized (Figure 2) [16].

### 3.2. Side Effects of Artificial Food Preservatives

Artificial food preservatives refer to chemical substances that are added to food products in order to limit bacterial growth and chemical alterations. The use of artificial food preservatives is intended to prolong the longevity of food items by impeding the proliferation of microorganisms, encompassing bacteria, yeast, and molds, which can cause spoilage and foodborne illnesses [18], in addition to inhibiting food oxidation reactions, especially by scavenging free radicals that may lead to food rancidity [19]. Hence, artificial food preservatives can help maintain the freshness, flavor, and overall quality of food products [16].

The era of agri-food industries is known for the introduction of various artificial food preservatives, including benzoates in acidic foods and beverages, sulfites to prevent browning and discoloration in fruits and vegetables, nitrites and nitrates to prevent the growth of harmful bacteria in meat products, propionates, which are used in the preparation of bread and other baked products, antioxidants to prevent the oxidation of fats and oils, etc. In fact, although there are beneficial effects from using artificial preservatives to extend the shelf life and maintain food quality, their widespread use has been accompanied by harmful side effects on human health [16].

**Figure 3:**
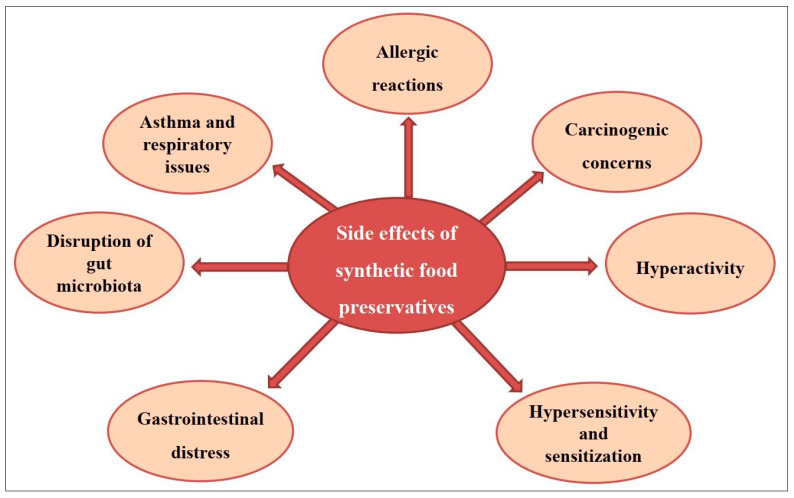
Possible side effects of artificial food preservatives.

In this regard, recent studies have targeted concerns about the side effects of artificial food preservatives, including allergic reactions, carcinogenic concerns, hyperactivity in children, gastrointestinal distress, asthma and respiratory issues, disruption of the gut microbiota, hypersensitivity, and sensitization [20,21] (Figure 3). Regulatory agencies such as the FDA (Food and Drug Administration) in the United States set limits on the use of these preservatives in foods to ensure they are safe for consumption. In addition, it was recommended that a safe daily intake should not exceed 5 milligrams per kilogram of body weight for sodium benzoate, which is widely used due to its antibacterial and anti-fungal properties [22]. However, recent research suggests that the combination of sodium benzoate and artificial colors in food can lead to hyperactivity [23]. This neurodevelopmental disorder affects millions of people, causing difficulties regarding time management and task completion. While there is no specific treatment for this disorder, affected individuals may benefit from medication and behavioral therapy. Additionally, it has been shown that artificial food preservatives may induce serious allergic reactions [24] and asthma [25], which could be considered triggers or aggravating factors in sensitive individuals. For example, sulfites can cause allergic reactions and asthma in some people, leading to symptoms such as hives, itching, difficulty breathing, and even anaphylaxis in severe cases [26]. Furthermore, some studies and animal trials have raised concerns about the potential carcinogenicity of certain artificial preservatives. For instance, there has been some debate over the safety of nitrites and nitrates in processed meats, as these compounds can form nitrosamines, which are potentially carcinogenic [27]. Moreover, artificial food preservatives may induce digestive discomfort, including symptoms such as bloating, gas, and diarrhea. A study has reported the correlation between carrageenan exposure and the manifestation of colonic ulcerations and gastrointestinal neoplasms in animal models [28]. Some studies have also suggested that certain artificial preservatives may have an impact on the composition of the gut microbiota, potentially affecting gut health. Based on in vitro studies, animal models, and human clinical trials, Cao et al. [29] reported that food additives could modify the gut microbiota and human health status. However, prolonged or high-level exposure to certain artificial food preservatives may lead to hypersensitivity or sensitization, whereby individuals become more reactive to the preservative over time [30].

### 3.3. Food Spoilage Microorganisms

In fact, food spoilage is a complex process biotic factors, such as microorganisms and enzymes, interact with abiotic factors such as temperature, humidity, light, and oxygen levels to influence food deterioration and spoilage. Maintaining optimal storage conditions and minimizing microbial contamination are crucial for preserving food quality and safety [31,32].

Spoilage, which is defined as “an organoleptic change in food”, can happen at any point in the food chain [33]. Insects, physical damage, native enzyme activity, and microbes can all contribute to the deterioration of foods. Indeed, food spoilage microorganisms are defined as various types of bacteria, yeasts, molds, and other microorganisms that can grow on or within food, leading to changes in its quality, flavor, texture, and appearance [3]. These microorganisms thrive in different conditions and can cause the spoilage of various types of foods. Psychrotrophic bacteria can grow at low temperatures and are responsible for spoilage in refrigerated foods such as dairy products and meats [34]. Thermophilic bacteria thrive at high temperatures and can cause spoilage in processed foods, canned products, and sauces [35]. Lactic acid bacteria contribute to the fermentation of foods but can also cause spoilage if they proliferate excessively, especially with some strains that possess proteolytic activity when in meat products [36]. Additionally, yeasts and molds are often responsible for spoilage in sweet products, including bread, fruits, vegetables, syrups, and dairy products [37,38].

The biochemical activity of microbial chemical processes has led to this complicated ecological phenomenon, which will ultimately be dominated by pre-existing ecological variables. When microbes multiply in food, they secrete enzymes that induce off-putting byproducts [39]. Metabolic processes in microbial food degradation yield substrates such as organic acids, esters, carbonyls, diamines, alcohols, sulfur compounds, hydrocarbons, and fluorescent pigments. The detection of microbial toxins or spores causing food contamination is difficult, as they often remain latent until a foodborne illness outbreak transpires, despite our reliance on chemical and physical attributes for microorganism spoilage detection. Hence, the intricate mechanisms and interplays driving food deterioration largely persist as subjects of significant obscurity [33]. This is despite the fact that food spoilage has substantial health and economic implications.

### 3.4. Natural Antimicrobial Agents

**Figure 4:**
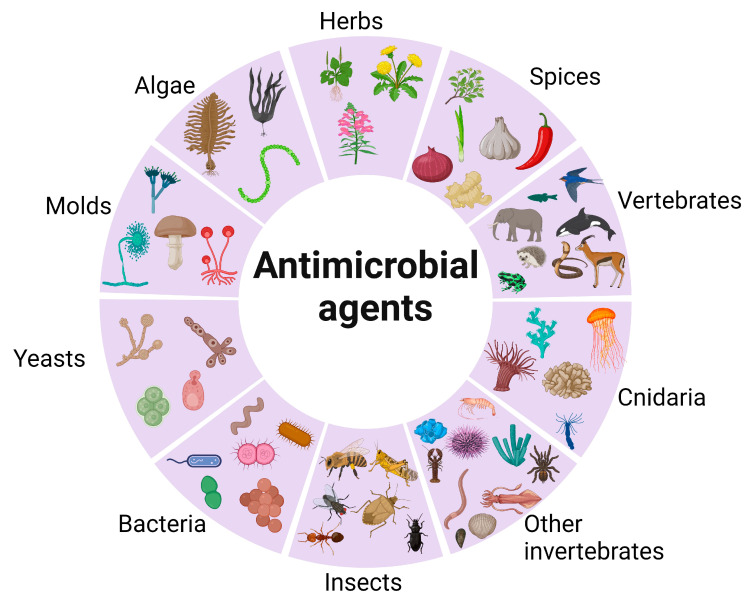
Diverse sources of antimicrobial agents from nature, involving either phytochemicals, peptides, or other molecules.

In the realm of contemporary medicine, the vast array of compounds found in nature serves as a rich source, with the potential to combat pathogens. This assumes paramount importance, particularly in the face of the growing crisis of antimicrobial resistance. Prominent origins of natural compounds endowed with valuable antimicrobial properties encompass fungi, bacteria, medicinal plants, marine organisms, and terrestrial organisms (Figure 4). Nevertheless, a substantial diversity of flora and fauna remains largely untapped, holding great potential for yielding further antimicrobial candidates and pharmaceutical agents when subjected to systematic exploration [40].

The increasing demand for chemical-free food and the limited efficacy of traditional food preservation methods in inhibiting foodborne pathogens have driven the adoption of antimicrobials by the food industry [41]. This emerging technology aims to prolong the shelf life of food products and address issues related to food quality and safety. Antimicrobials can be either natural or synthetic, with a growing emphasis on the importance of natural antimicrobials over their synthetic counterparts. Despite the approval of synthetic preservatives for human consumption given by government organizations, concerns persist regarding their potential adverse effects on health [42].

Various sources provide natural antimicrobials that are employed to safeguard food against spoilage and pathogenic microorganisms. Plants, including herbs, spices, fruits, vegetables, seeds, and leaves, constitute the primary reservoir of antimicrobial compounds, many of which contain essential oils with potent antimicrobial properties [43]. Notably, herbs and spices such as rosemary, sage, basil, oregano, thyme, cardamom, and clove are abundant in essential oils. These oils play a crucial role in enhancing food quality and extending shelf life by efficiently combating a wide range of pathogenic and spoilage microorganisms, including *Salmonella* spp., *Escherichia coli*, *Listeria monocytogenes*, *Campylobacter* spp., and *Staphylococcus aureus* [44,45]. Furthermore, these antimicrobial substances find applications as edible food coatings, which effectively prevent the proliferation of bacteria on food and the surfaces of food-related products.

Antimicrobial activity is of paramount importance in modern medicine, referring to the ability of a substance to eliminate or impede the growth of microorganisms such as bacteria, viruses, fungi, and protozoa. These antimicrobial substances find widespread applications in areas such as food preservation, medicine, and personal hygiene. The advent of antimicrobial drugs has heralded groundbreaking advancements in the treatment of infectious diseases, leading to the preservation of numerous lives [46].

Antimicrobial compounds can be synthesized in laboratories or may be found naturally in microorganisms, animals, and plants. Throughout history, the natural antibacterial chemicals present in various herbs and spices have been utilized to preserve food freshness and prevent spoilage. Furthermore, certain invertebrates and mammals produce antimicrobial peptides, which serve as defense mechanisms against diseases.

### 3.5. Spices as a Source of Natural Antimicrobial Agents

Spices refer to the aromatic components derived from various plant parts such as seeds, fruits, roots, barks, or other plant substances, and are primarily employed for enhancing the flavor or adding color to food. These substances are distinct from herbs, which comprise the leaves, flowers, or stems of plants and serve different culinary purposes [47]. The Food and Drug Administration (FDA) defines spices as “whole, broken, dried, or ground vegetable substances used for flavoring and seasoning food” [48].

Throughout ancient and medieval culinary history, spices have played a pivotal role, with their utilization influenced not only by taste preferences but also by prevailing medical theories concerning diet and health. Spices have a rich history of traditional medicinal use for treating a wide range of ailments. In recent decades, numerous preclinical and clinical studies have provided compelling evidence supporting the beneficial effects of spices and their active components in preventing and managing various diseases, such as diabetes, arthritis, cancer, asthma, cardiovascular diseases, and neurodegenerative diseases [49]. Spices emanate from desiccated botanical components such as flower buds and blooms (e.g., cloves and saffron), subterranean rhizomes (ginger and turmeric), inner bark (cinnamon), reproductive structures such as fruits and berries (e.g., cloves, chili, and black pepper), or embryonic propagules (e.g., cumin) [50]. They may also include various herbs such as marjoram, parsley, mint, rosemary, oregano, and thyme [51]. The spice trade has evolved into a significant economic activity due to its favorable characteristics and diverse applications. In 2020, spice exports were valued at USD 3.61 billion, experiencing a 23.2% increase from 2019. In terms of global trade, spices account for 0.022% [52]. Spice sales are increasing annually worldwide and modern food production and trade globalization have made spices readily available year-round in developed nations [53]. Spices have demonstrated significant antimicrobial properties, which make them valuable in countering microbial infections. Their active constituents often exhibit natural antimicrobial properties that help to inhibit the growth and proliferation of various pathogenic microorganisms, such as bacteria, fungi, and even some viruses. Moreover, due to their inherent ability to inhibit the growth of spoilage-causing microorganisms, spices have been utilized as natural food preservatives since ancient times [54].

### 3.6. Suggested Antimicrobial Spices for Food Preservation

Quantifying the precise number of spices in the world proves challenging, due to the influence of multi-cultural and culinary practices. Based on the existing literature, an exact count of the spices that are currently employed remains elusive. Nonetheless, it is estimated that there are roughly 112 types of spices in use worldwide today [55]. The present review has identified a restricted selection of 25 spice types that exhibit significant antimicrobial properties (Table 1). Given the diverse array of chemical compound groups present in herbs and spices, it is probable that their antimicrobial activity does not hinge on a singular mechanism. Instead, multiple mechanisms likely come into play, each targeting various aspects of microbial cells. Nevertheless, these various mechanisms can generally be categorized into two overarching objectives: impeding the proliferation of spoilage microorganisms to facilitate food preservation, and restraining or modulating the growth of pathogenic microorganisms [11,56]. Our study also introduces these spices (in brief) and their active constituents within the realm of food science, suggesting the possibility of exploring their potential as a viable alternative to artificial preservatives for food preservation purposes.

#### 3.6.1. Allspice

*Pimenta dioica* (L.) Merr. (allspice), a fragrant botanical belonging to the Myrtaceae family, attains a height of up to 20 m and a diameter of approximately 30 cm. Native to regions including Guatemala, Southern Mexico, Central America, Belize, and Antilles, this plant’s fruit, measuring 4–8 mm in diameter, emanates a highly aromatic essence and serves as a widely utilized spice. The name ‘allspice’ in English originates from its distinctive ability to amalgamate the distinct flavors of cloves, nutmeg, cinnamon, and black pepper [57]. It is advisable to consider the potential utilization of the abundant bioactive compound, eugenol, which is found in *Pimenta dioica*, as a plant-based food preservative. This recommendation is rooted in its demonstrated antioxidant and antifungal properties, as well as its proven efficacy within food systems [58]. The antimicrobial efficacy of allspice essential oil and its predominant compound, eugenol, was investigated against a diverse collection of Gram-negative and Gram-positive bacteria associated with foodborne illnesses. The study revealed substantial antibacterial activity, along with a noteworthy capacity to impede quorum sensing (QS)-dependent mechanisms and suppress biofilm formation, even at low concentrations [59].

#### 3.6.2. Anise

*Pimpinella anisum* L. (anise) is a widely distributed herbaceous medicinal plant of the Apiaceae family, prominently employed as a spice across tropical, subtropical, and temperate regions globally. The fruits possess inherent aromatic qualities, which can be attributed to the presence of sulfur-containing compounds, terpenes, and minor constituents such as anisaldehyde and estragol, thus rendering it an ideal flavoring agent for various food products, including bread, cakes, and biscuits [60]. Analysis of anise revealed the presence of 11 distinct identified components, collectively constituting 95.11% of the total oil content. Notably, the major compound was found to be anethole (51.02%), followed by estragole (24.75%) and fenchone (13.22%) [61]. The essential oil derived from anise seeds demonstrated noteworthy antimicrobial potency against a diverse array of bacteria, encompassing both Gram-positive and Gram-negative pathogens, as well as yeasts. Particularly significant inhibition was observed against *Pseudomonas aeruginosa* (109.84%), *Staphylococcus aureus* (41.21%), and *Candida albicans* (72.04%) [62]. It has been documented that applying a coating infused with anise (*Pimpinella anisum*) essential oil and ginger extract to beef samples, which are then packaged in a modified atmosphere, effectively mitigated microbial, chemical, and sensory deterioration. This intervention successfully extended the shelf life of fresh beef under modified atmospheric packaging conditions and suggests its use as a natural food preservative [63].

#### 3.6.3. Basil

Basil (*Ocimum basilicum* L.), a member of the Lamiaceae family, is renowned for its rich flavoring properties and finds versatile applications as a culinary, medicinal, and ornamental spice. Traditionally, basil has been employed as a medicinal plant, with historical uses encompassing the treatment of various ailments such as headaches, coughs, diarrhea, constipation, warts, worms, and kidney malfunctions [64]. In a published study, the chemical compositions of freshly harvested basil leaves were subjected to analysis using gas chromatography–mass spectrometry (GC-MS), which successfully identified 11 distinct components. The predominant constituents were identified as methyl cinnamate (70.1%), linalool (17.5%), β-elemene (2.6%), and camphor (1.52%) [65,66]. The essential oil of basil demonstrated significant antimicrobial efficacy, showing high potential against pathogenic microorganisms such as *Staphylococcus epidermidis* (MTCC 435), *Streptococcus mutans* (MTCC 890), *Escherichia coli* (MTCC 723), *Candida albicans* (ATCC 14053), and *Candida kefyr* (ATCC 204093) [67]. Basil, with its distinctive bioactive composition, has a historical culinary tradition of serving as a preservative agent [68].

#### 3.6.4. Bell Pepper

*Capsicum annuum* L., commonly referred to as bell pepper, red pepper, or chili, pertains to the botanical family Solanaceae. This plant species is cultivated in diverse geographical areas, including subtropical, tropical, and temperate regions in Africa, Asia, the Americas, and Mediterranean countries. In addition to its considerable dietary importance, bell pepper has been the subject of extensive research in recent years, revealing various pharmacological properties such as anti-inflammatory, antioxidant, cardioprotective, antimicrobial, and anticarcinogenic activities [69]. The fruits of *Capsicum annuum* L. harbor capsaicinoids, which are a group of bioactive compounds responsible for conferring their characteristic pungent taste. Among these capsaicinoids, capsaicin and dihydrocapsaicin stand out as the primary components, contributing to approximately 90% of the overall intense piquant sensation found in pepper fruits [70]. Numerous investigations have demonstrated the antimicrobial properties of diverse bell pepper extracts against a broad spectrum of foodborne pathogens. These pathogens include, but are not limited to, *Listeria monocytogenes*, *Staphylococcus aureus*, *Escherichia coli*, *Salmonella typhimurium*, *Bacillus subtilis*, *Proteus mirabilis*, *Lactobacillus acidophilus*, and *Lactobacillus plantarum*. Additionally, bell pepper extracts have been found to exhibit antifungal activity against *Cochliobolus* spp. [71]. Considering the antimicrobial and antioxidant attributes found in bell-pepper-derived products, investigations have explored their potential utility as antimicrobial agents for food preservation, with the aim of managing foodborne pathogens and mitigating product spoilage [72].

#### 3.6.5. Black Pepper

Black pepper (*Piper nigrum* L.) is known as the king of spices. It is widely used as a spice worldwide and has various applications, including medicinal and preservative uses. Piperine, its active compound, shows physiological effects, such as antioxidant and antidiarrhoeal properties, and enhances digestion. It also enhances drug bioavailability and exhibits non-genotoxic, anti-mutagenic, and anti-tumor properties [73]. Black pepper was found to be rich in aromatic compounds, polyphenols, flavonoids, alkaloids, amides, and lignans [74]. The primary compound of black pepper is piperine [75]. The antibacterial activities of piperine were investigated against a group of Gram-positive bacteria, including *Staphylococcus aureus*, *Staphylococcus epidermidis*, *Bacillus subtilis*, *Enterococcus faecalis*, and Gram-negative bacteria, such as *Salmonella enterica*, *Klebsiella pneumonia*, and *Escherichia coli.* Piperine exhibited significant antibacterial activity against all tested strains, with a minimum inhibitory concentration (<325 μg/mL) [76]. Furthermore, the essential oils of black pepper exhibited notable antifungal effectiveness against *Aspergillus flavus* [77]. Black pepper is proposed as a natural food preservative due to its antimicrobial and antioxidant properties. Notably, its antioxidant capacity rivals that of synthetic antioxidants such as butylated hydroxyanisole (BHA) and butylated hydroxytoluene (BHT), which are commonly employed in food preservation. Consequently, naturally occurring antioxidants may surpass BHA and BHT in terms of their capability to neutralize mutagens in food [78].

#### 3.6.6. Black Seeds

The seeds of *Nigella sativa* L., also known as black seeds or black cumin, have a rich history of traditional use as a condiment and remedy for various ailments across different cultures and countries. Islamic, Unani, African, Eastern, Middle Eastern, Chinese, and Ayurvedic medicines frequently describe and recommend this plant for treating diverse diseases. Scientific studies have demonstrated its efficacy in managing diabetes, hypertension, oxidative stress, epilepsy, ulcers, asthma, inflammatory disorders, fatty liver, cancers, and arthritis in both model organisms and human subjects [79]. It was reported that, as per GC-MS analysis, the predominant constituents of black seed oil were found to be caryophyllene (17.47%), followed by thymoquinone (11.80%), 1,4-cyclohexadiene (7.17%), longifolene (3.5%), and carvacrol (1.82%). Thymoquinone, known as the most bioactive ingredient in black seeds, exhibited a concentration of 6.63 mg/mL in oil extracted using supercritical fluid extraction, and 1.56 mg/mL in oil obtained through the cold press method [80]. The antibacterial activity of the black seeds extracts was assessed against various bacteria, including *Escherichia coli*, *Klebsiella pneumoniae Staphylococcus aureus*, *Enterobacter aerogenes*, and *Fusarium solani*, exhibiting various levels of antimicrobial efficacy [81]. Thymoquinone, derived from black seeds, demonstrated noteworthy antifungal activity against various fungi, including *Candida albicans* AUMC 1299, *Aspergillus flavus* AUMC 1276, *Fusarium oxysporum* AUMC 215, *Scopulariopsis brevicaulis* AUMC 1653, *Geotrichum candidum* AUMC 226, and *Trichophyton rubrum* AUMC 1804 [82]. Black seeds have a historical lineage of use as food additives and flavorings. In contemporary contexts, their aromatic oils find application in various rural industries, encompassing food preservation among other uses [83].

#### 3.6.7. Caraway

Caraway seeds (*Carum carvi* L.), which are renowned for their aromatic properties, have a long history of use as both a spice and a medicinal herb in Asia, Africa, and Europe. Folk medicine has employed caraway for diverse therapeutic purposes. Scientific investigations have revealed that caraway contains numerous bioactive metabolites, imparting a wide range of pharmacological effects, including antimicrobial, anticancer, antioxidant, hypolipidemic, antidiabetic, analgesic, diuretic, gastrointestinal, and bronchial relaxant activities, among others [84]. The major constituents of caraway seed oils are carvone (44.5–95.9%) and limonene (1.5–51.3%). A strong negative correlation of −0.999 (*p* < 0.01) was observed between the contents of carvone and limonene. Additionally, trace amounts of β-myrcene (0–0.4%), trans-dihydrocarvone (0–0.5%), and trans-carveole (0–0.2%) were identified, along with α-pinene, sabinene, n-octanal, trans-β-ocimene, cis- and trans-limonene oxide, γ-terpinene, linalool, cis-dihydrocarvone, perillaldehyde, trans-anethole, cis-carveol, and trans-β-caryophyllene in the caraway oils [85,86]. The observed zone of inhibition for a starch bio-based composite active edible caraway essential oil against all tested foodborne bacteria exhibited a remarkably high level of significance (*p* < 0.01) when compared to the control group. Additionally, it was noteworthy that the antibacterial activity against biofilms was more pronounced against Gram-positive bacteria in comparison to Gram-negative bacteria [87]. Moreover, the essential oils of caraway seeds exhibited remarkable antifungal activity against *Aspergillus flavus*, in addition to a degree of antiaflatoxinogenic potential, making it a compelling candidate for consideration as a food preservative [88].

#### 3.6.8. Cardamom

Cardamom (*Elettaria cardamomum* (L.) Maton), commonly known as small cardamom, green cardamom, or true cardamom, is cultivated in various countries, including India, Guatemala, Sri Lanka, Nepal, Indonesia, Costa Rica, Mexico, and Tanzania. Throughout history, cardamom capsules have found diverse culinary and traditional medicinal applications, such as managing asthma, dental and gum infections, digestive and kidney disorders, cataracts, nausea, diarrhea, and cardiac disorders. The distinctive aroma and functional properties of cardamom capsules can be attributed to the presence of essential oils and other bioactive metabolites, making them valuable for use in food and the pharmaceutical field [89]. Cardamonin, a chalconoid that naturally occurs in cardamom spice, has been identified in other plants such as *Alpinia katsumadai* and *Alpinia conchigera* through various research studies. Its multiple potential health benefits have sparked increasing interest among researchers in the scientific community [90]. Moreover, the hydrodistilled essential oil obtained from both the seed and fruit coat of *Elettaria cardamomum* (L.) Maton, growing in South India, was analyzed using GC and GC-MS. A total of twenty-five constituents were identified, constituting 95.28% and 96.58% of the oil from the seed and fruit coat, respectively. Their major constituents, including limonene (4.05% and 3.82%), 1,8-cineole (15.13% and 23.74%), α-terpineol (4.67% and 5.25%), and α-terpinyl acetate (56.87% and 51.25%), were found to be common in both the seed oil and fruit coat oil, albeit in varying proportions [91]. The ethanol extract of cardamom demonstrated notable antibacterial efficacy against *Bacillus subtilis* and *Staphylococcus aureus*, as well as *Pseudomonas aeruginosa*. However, it is important to note that no antibacterial activity was detected against *Escherichia coli* [92]. Moreover, the ethanolic extract of cardamom demonstrated noteworthy antibacterial efficacy against a panel of foodborne pathogenic strains, including methicillin-resistant *Staphylococcus aureus* ATCC 43300, *Staphylococcus aureus* ATCC 29213, *Salmonella typhimurium* ATCC 14023, *Pseudomonas aeruginosa* ATCC 9027, and *Escherichia coli* ATCC 25922. These findings substantiate the potential utilization of these extracts in the formulation of bio-preservatives for food safety, circumventing the undesirable side effects associated with artificial preservatives [93].

#### 3.6.9. Cinnamon

Cinnamon, a tropical tree belonging to the Lauraceae family, encompasses approximately 250 species, with four of them being of significant commercial importance and traded globally. The dried inner bark of cinnamon, particularly *Cinnamomum verum* J. Presl, has been employed as a spice, flavoring agent, and food seasoning for thousands of years. Additionally, it holds a prominent place in traditional medicine, where it is used to treat various conditions, including diabetes, toothache, tumors, diarrhea, fever, common cold, nausea, flatulence, amenorrhea, headache, cough, cardiovascular diseases, eye inflammation, bad breath, rheumatism, dyspnea, leukorrhea, frigidity, vaginitis, impotency, and neuralgia [94]. Cinnamaldehyde and trans-cinnamaldehyde are the principal constituents found in cinnamon essential oil, responsible for its characteristic fragrance and diverse range of observed biological activities [95]. The essential oils of *Cinnamomum verum* J. Presl showed broad-spectrum antimicrobial efficacy against various microorganisms, including Gram-positive and Gram-negative bacteria, yeasts, and molds. The tested microorganisms included *Micrococcus luteus*, *Bacillus cereus*, *Bacillus subtilis*, *Klebsiella aerogenes*, *Salmonella enterica*, *Escherichia coli*, *Candida albicans*, *Candida tropicalis,* and *Penicillium expansum*. The results exhibited low MIC, MBC, and MFC values, suggesting potential bactericidal and fungicidal effects [96]. Cinnamon, one of the most ancient spices, has a historical tradition of serving as a natural preservative in the realms of the food, beverage, and cosmetic industries [97].

#### 3.6.10. Clove

The aromatic flower buds of *Syzygium aromaticum* (L.) Merr. & L.M. Perry, commonly referred to as clove, holds a prominent status as one of the most valuable spices and is known for its extensive utilization throughout history, particularly in Sri Lanka, dating back to approximately 900–1100 CE. Its diverse array of biological and therapeutic applications includes serving as an antioxidant, antimicrobial, antifungal, antiviral, and analgesic agent. Additionally, it contributes to digestion, provides relief from tooth pain, and exerts a notable influence on the reproductive system. Furthermore, clove plays a crucial role in agriculture as a larvicidal agent [98]. Clove is a fragrant spice; eugenol constitutes the predominant compound in clove essential oil, constituting no less than 50% of its composition, while the remaining 10–40% comprises eugenyl acetate, α-humulene, and β-caryophyllene. The principal biological activities associated with these compounds are outlined in the study. Additionally, this essential oil has found significant application within the food industry, with recent research demonstrating that its incorporation into baked and processed foods can effectively prolong shelf life while preserving the original taste, flavor, texture, appearance, and overall sensory acceptability [99]. The antimicrobial potential of essential oil from clove exhibited varying degrees of efficacy against *Staphylococcus aureus*, *Listeria innocua*, and *Pseudomonas aeruginosa*. Scanning electron microscopy (SEM) analysis revealed a pronounced impact of the essential oil on the integrity of cell membranes, potentially resulting in cytoplasmic leakage and subsequent cell death [100]. The antimicrobial effectiveness of clove also demonstrated a broad spectrum of activity, with inhibition zones exceeding 10 mm and MIC values ranging from 112.13 to 64.25 µL/mL against a range of microorganisms, including *Serratia marcescens*, *Staphylococcus aureus*, and *Bacillus subtilis*, as well as various Penicillium species, such as *Penicillium commune*, *Penicillium expansum*, *Penicillium glabrum*, and *Penicillium chrysogenum* [101]. In addition, previous studies indicate that clove exhibits antioxidant capabilities comparable to those of butylated hydroxytoluene (BHT), a prevalent synthetic antioxidant utilized in food preservation. This suggests the potential for clove to serve as a natural preservative, enhancing oxidative stability, antioxidant attributes, flavor, shelf life, and food coloration [102].

#### 3.6.11. Coriander

The seeds of coriander (*Coriandrum sativum* L.), one of the oldest spices employed by human civilization, is believed to have originated in the Middle East and the Mediterranean regions, potentially spreading to various parts of the world, including China, Europe, India, Africa, and Asia. Despite its widespread use, the exact origins of coriander remain uncertain. Both its seeds and leaves serve as valuable spices. Throughout history, coriander seeds have been traditionally utilized for diverse medicinal purposes, including relieving pain, easing the symptoms of rheumatoid arthritis, possessing anti-inflammatory properties, treating mouth ulcers and eye redness, alleviating gastrointestinal disorders, and even aiding in the regulation of blood glucose levels [103]. The analysis of the essential oil extracted from coriander seeds revealed its chemical composition, wherein linalool emerged as the predominant component, constituting approximately 57.57% of the oil. Subsequently, geranyl acetate accounted for 15.9% of the composition, followed by beta-caryophyllene (3.26%), camphor (3.02%), and p-cymene (2.5%). Additionally, trace amounts of various other phytochemicals were detected in the oil [104]. As reported in a previous publication, coriander essential oil exhibited significant antibacterial activity against *Bacillus subtilis*, with the subsequent notable effect observed against *Stenotrophomonas maltophilia*, derived from the milk industry. Additionally, the essential oil of coriander displayed remarkable antifungal activity, particularly against *Penicillium expansum* isolated from grapes [105]. The antimicrobial activity of essential oils from *Coriandrum sativum* L. seeds was evaluated against specific foodborne pathogens, including *Streptococcus pyogenes*, *Listeria monocytogenes*, *Bacillus subtilis*, *Enterobacter aerogenes*, *Salmonella typhimurium*, and *Shigella dysenteriae.* The findings demonstrated the presence of significant antibacterial activity, implying coriander’s potential as a food preservative when combined with essential oils from *Cuminum cyminum* L. [106].

#### 3.6.12. Cumin

Cumin (*Cuminum cyminum* L.) is a widely recognized and esteemed spice with a long history of culinary usage worldwide. The spice possesses a potent, slightly bitter, and pungent flavor. Traditionally, cumin has been employed for various medicinal purposes, including its anti-inflammatory properties, diuretic effects, and its ability to alleviate gas and muscle spasms. It has been utilized as an aid for indigestion, jaundice, diarrhea, and flatulence, and is antiseptic and antihypertensive, with applications ranging from poultices and suppositories to oral consumption. Furthermore, cumin has found its place as a fragrant component in the formulation of creams, lotions, and perfumes [107]. Cumin’s composition includes the volatile oil, constituting approximately 3–4% of its content. The principal active component is cuminaldehyde, which is notably abundant, representing around 45–50% of the total oil content. Additionally, cumin contains various other compounds that are present in smaller quantities, including cuminic alcohol, p-mentha-1,3-dien-7-al, γ-terpinene, p-mentha-1,4-dien-7-al, p-cymene, perillaldehyde, and β-pinene [108]. The essential oil extracted from *Cuminum cyminum* L. demonstrated significant antibacterial efficacy against various food-borne pathogens, including *Bacillus cereus*, *Staphylococcus aureus, Salmonella typhi,* and *Escherichia coli.* Among these pathogens, the essential oil exhibited the most potent antimicrobial activity against *Bacillus cereus*, with an inhibition zone comparable to that of tetracycline, a well-known antibiotic [109]. An investigation into the antifungal properties of *Cuminum cyminum* L. essential oil against three postharvest fungal pathogens, namely, *Botrytis cinerea*, *Penicillium expansum,* and *Aspergillus niger*, demonstrated that concentrations of ≥750 µL/L resulted in the complete inhibition of mycelial growth in the tested fungi. These findings suggest that the essential oil derived from this specific cumin chemovar holds promise as a potential natural food preservative in various applications [110].

#### 3.6.13. Dill

Dill (*Anethum graveolens* L.) is an exclusive member of the Anethum genus and the seeds and leaves have a rich history of utilization in Ayurvedic medicine, dating back to ancient times. This popular herb finds widespread use, both as a culinary spice and as a source of essential oil. Both the aerial parts and the seeds of dill are employed for various purposes. Notably, the aromatic dill seeds possess a distinct fragrance. The dill seeds offer several beneficial properties, including aromatic, carminative, mildly diuretic, galactogogue, stimulant, and stomachic effects. The essential oil present in the seeds is known to alleviate intestinal spasms and griping, providing relief from colic. Additionally, the carminative volatile oil enhances appetite, alleviates gas, and aids in digestion. Moreover, chewing the seeds aids in combatting bad breath [111]. Dill seeds’ essential oils were subjected to thorough analysis using gas chromatography (GC) and gas chromatography–mass spectrometry (GC/MS). The primary constituents identified in the fruit oil were found to be carvone (33.57%), myristicin (24.21%), limonene (15.02%), dihydrocarvone (13.13%), and carvacrol (4.92%). However, it is crucial to note that the percentage composition of dill’s constituents may be influenced by various factors, such as environmental conditions and variations in the analysis process [112]. Dill oil exhibited robust antimicrobial activity against *Staphylococcus aureus*, *Yersinia enterocolitica*, *Escherichia coli*, *Rhodotorula glutinis*, and *Geotrichum candidum*, displaying inhibition zones ranging from 36 to 69 mm. Furthermore, it demonstrated moderate effectiveness against *Salmonella typhimurium*, with an inhibition zone of 26 mm. In addition to its antibacterial properties, dill oil also demonstrated significant antifungal activity against *Candida albicans* and *Saccharomyces cerevisiae*. However, its effectiveness against molds, such as *Aspergillus niger*, was found to be more modest in comparison [113]. Dill possesses a pleasing and piquant fragrance. Both the essential oils extracted from dill seeds and from the leaves are widely employed as flavor enhancers in food and beverages. Scientific investigations have demonstrated the antimicrobial, antifungal, and antioxidant properties of dill and its essential oil. Hence, they are recommended as viable and secure alternatives to synthetic preservatives in food applications [114].

#### 3.6.14. Fennel

The seeds of *Foeniculum vulgare* Mill., commonly known as fennel, constitute a widely distributed spice known for its aromatic fragrance. Originally native to Southern Europe and the Mediterranean region, fennel is now cultivated extensively across temperate and tropical regions worldwide. In traditional medicine, fennel has been utilized to address various conditions such as rheumatism, cold-related pain, and digestive issues. Recent scientific studies have highlighted its potential as an effective analgesic, anti-inflammatory, and antioxidant agent. Additionally, fennel has demonstrated promising antimicrobial and antiviral properties. These findings underscore its potential significance as a valuable natural remedy with diverse health benefits [115]. Gas chromatography/mass spectrometry (GC/MS) analysis revealed that phenylpropanoids constituted the predominant group of compounds present in the essential oil. Among these, anethole stood out as the most abundant, accounting for 75.8% of the composition. In general, *Foeniculum vulgare* Mill. is characterized by high anethole content. Additionally, minor components, including limonene (5.67%), methyl chavicol (4.56%), p-anisaldehyde (3.99%), and fenchone (2.82%), were discerned in trace amounts [116]. Another research study revealed that the chemical compositions of fennel leaves exhibited variability among the different varieties. Nevertheless, across various fennel varieties, the t-anethole content consistently ranks highest, followed by phenolic compounds and flavonoid compounds, respectively [117]. Fennel seed-derived essential oil demonstrated significant antibacterial efficacy against specific foodborne pathogens, namely, *Staphylococcus albus*, *Bacillus subtilis*, *Salmonella typhimurium*, *Shigella dysenteriae*, *and Escherichia coli*, as determined from low MIC and MBC values. Notably, among these bacterial strains, *Shigella dysenteriae* exhibited the highest susceptibility to the essential oil of fennel and was suggested as a food preservative [118]. Moreover, fennel essential oils exhibited noteworthy antimicrobial efficacy against a diverse array of bacterial and fungal strains, encompassing Gram-negative bacteria such as *Salmonella typhi* ATCC 13076 and *Escherichia coli* O157 ATCC 1659, as well as Gram-positive bacteria, including *Bacillus cereus* ATCC 11778 and *Staphylococcus aureus* ATCC 13565. Furthermore, the essential oils demonstrated antimicrobial activity against the yeast *Candida albicans* ATCC 10231 and the mold *Aspergillus flavus* ATCC 16875 [119].

#### 3.6.15. Fenugreek

The seeds of *Trigonella foenum-graecum* L., commonly known as fenugreek, hold historical significance as an esteemed antiquated spice, with documented references dating back to 1500 BCE in the Ebers Papyrus from Egypt. Its natural habitat encompasses Asia and the Mediterranean region. Fenugreek exhibits diverse potential therapeutic applications, including alleviating conditions such as head colds, catarrh, influenza, constipation, bronchial issues, diabetes, emphysema, asthma, pneumonia, tuberculosis, hay fever, sore throat, pleurisy, laryngitis, sinusitis, and fostering lactation [120]. Previous studies stated that the phytochemical evaluation of fenugreek has unveiled a diverse spectrum of secondary metabolite classes, encompassing saponins, steroids, flavonoids, alkaloids, terpenes, phenolic acid derivatives, fatty acids, and amino acids, along with their respective derivatives. Additionally, research investigations have elucidated that the predominant phytochemical constituent within fenugreek is sotolone, serving as the principal inducer of its distinctive “seasoning flavor.” Furthermore, it has been demonstrated that sotolone exhibits the capacity to effectively counteract oxidative stress within pancreatic cells [121]. The fenugreek seeds displayed notable antimicrobial efficacy, demonstrating varying degrees of activity against *Staphylococcus aureus* (ATCC 25923), *Bacillus subtilis* (NCTC 8236), *Escherichia coli* (ATCC 25922), *Pseudomonas aeruginosa* (ATCC 27853), as well as *Candida albicans* (ATCC 7596) and *Aspergillus niger* (ATCC 9763) [122]. The ethanolic extract derived from fenugreek was blended with green tea extract and subsequently incorporated into a chitosan coating applied to Pacific white shrimp, and the experimental findings revealed that this composite coating effectively prolonged the shelf life of the shrimp and led to noteworthy improvements in various quality indicators, including total volatile bases, nitrogen, thiobarbituric acid-reacting substances, total bacterial count, and pH levels. This composite coating is recommended as a valuable food preservative [123].

#### 3.6.16. Garlic

The bulbs of garlic, *Allium sativum* L., have garnered widespread recognition, both as a highly regarded culinary spice and as a popular remedy for diverse physiological ailments. With its origins traced back to central Asia, garlic gradually disseminated to regions including China, the Near East, and the Mediterranean, and eventually extended westward to Central and Southern Europe, Northern Africa, and Mexico. Over millennia, garlic has maintained a role in medicinal applications. Presently, garlic’s predominant medicinal applications encompass its capacity to mitigate and manage cardiovascular conditions by reducing blood pressure and cholesterol levels, in addition to its recognized roles in anticancer, antiatherosclerosis, and hypolipidemic interventions [124]. The bulbs of *Allium sativum* L. are known to harbor a plethora of phytochemical compounds. Predominantly, allicin stands out as the major constituent, although the bulbs also contain a spectrum of other sulfur-containing compounds, including ajoenes, vinyldithiins (specifically, 2-vinyl-(4H)-1,3-dithiin and 3-vinyl-(4H)-1,2-dithiin), sulfides (such as diallyl disulfide and diallyl trisulfide), and various additional constituents [125]. A scientific investigation explored the potential utility of garlic as a food preservative. The study revealed that both freeze-dried fresh garlic and spray-dried microencapsulated garlic essential oil, when employed at a 20% concentration, exhibited significant antimicrobial properties. This finding suggests a promising application for these garlic-based preservation methods in food systems, particularly in the preservation of meat and meat products when stored within the temperature range of 4–8 °C [126]. The outcomes of a published study employing the disc-diffusion method for in vitro antibacterial analysis have demonstrated the potent antibacterial efficacy of garlic against a broad spectrum of tested bacteria, effectively competing with the antibiotic Chloramphenicol. Notably, its effectiveness was particularly pronounced against Gram-positive bacteria. Among the Gram-positive strains, the most susceptible were *Staphylococcus epidermidis* (with an inhibition zone of 27.5 ± 4.5 mm), followed by *Staphylococcus aureus* (20.5 ± 0.5 mm), *Staphylococcus saprophyticus* (20.5 ± 0.5 mm), *Bacillus cereus* (18.5 ± 0.5 mm), and *Streptococcus pneumoniae* (15.0 ± 1.0 mm). In the context of Gram-negative bacteria, *Escherichia coli* (14.0 ± 0.0 mm) and *Shigella flexneri* (14.0 ± 1.0 mm) exhibited the highest susceptibility, followed by *Proteus vulgaris* (12.5 ± 0.5 mm) and *Klebsiella pneumoniae* (10.0 ± 1.0 mm). Notably, the bacterium *Pseudomonas aeruginosa* did not demonstrate any susceptibility toward garlic juice [127]. Additionally, the literature highlights the antifungal capabilities of garlic against specific fungal strains, notably including *Penicillium citrinum*, *Aspergillus versicolor*, *Penicillium expansum*, and *Candida albicans* [128].

#### 3.6.17. Ginger

The rhizome of *Zingiber officinale* Roscoe, commonly referred to as ginger, yields a renowned spice that has enjoyed cultivation for millennia, serving both culinary and medicinal roles. Its origins trace back to Southeast Asia, with present-day India and China emerging as principal contributors to the global market supply. An array of studies substantiates its therapeutic significance in addressing a spectrum of conditions, encompassing diabetes, obesity, diarrhea, allergies, pain management, fever reduction, rheumatoid arthritis, cancer treatment, and tumor management. In addition to these properties, ginger exhibits notable antimicrobial and antioxidant attributes [129]. Gingerols represent the predominant pungent constituents within the rhizomes of ginger, and they are widely recognized for their substantial influence on human health and nutrition. It is noteworthy that gingerol analogs are highly susceptible to thermal instability, readily undergoing dehydration reactions to transform into shogaols, which are responsible for the distinct pungent flavor found in dried ginger. Both gingerols and shogaols are responsible for numerous biological activities [130]. A study aiming to encapsulate the chemical makeup of diverse ginger varieties highlighted the presence of 194 varieties of volatile oils, 85 varieties of gingerol, and 28 varieties of diarylheptanoid compounds within *Zingiber officinale* Roscoe [131]. Elsewhere, ginger exhibited comprehensive antibacterial efficacy encompassing a wide range of Gram-positive and Gram-negative bacterial strains. Notably, its activity extended to bacteria such as *Staphylococcus epidermidis*, *Staphylococcus aureus*, *Enterococcus faecalis*, *Streptococcus faecalis*, *Bacillus cereus*, *Bacillus megaterium*, *Escherichia coli*, *Bacillus subtilis*, *Klebsiella pneumoniae*, *Salmonella typhimurium*, *Salmonella typhi*, *Pseudomonas aeruginosa*, and various species of the genus *Proteus* [132]. Fungi are recognized for their exceptional resilience as spoilage microorganisms, often surpassing the control measures implemented by the food industry. Notably, essential oils extracted from ginger exhibited noteworthy antifungal efficacy against a wide spectrum of fungal strains, including but not limited to *Aspergillus niger*, *Aspergillus flavus*, *Penicillium expansum*, *Alternaria alternata*, *Fusarium oxysporum*, *Mucor hemalis*, *Penicillium notatum*, *Candida albicans*, and *Fusarium oxysporum*. Consequently, these findings underscore the strong potential of ginger essential oils as a highly recommended food preservative [133].

#### 3.6.18. Mastic

*Pistacia lentiscus* L., known by its common name mastic, enjoys extensive distribution throughout the Mediterranean basin and circum-Mediterranean regions. The production of mastic resin, also referred to as gum, is achieved through the exudation process. The desiccated mastic resin serves dual roles, being both a prevalent culinary spice and also demonstrating medicinal properties. Its historical roots can be traced back to the era of ancient Egyptian civilization, as evidenced by its presence in ancient Egyptian mummies. Scientific studies on mastic resin have illuminated a range of valuable attributes. These encompass antimicrobial, antioxidant, anticancer, anti-inflammatory, cardioprotective, and wound-healing activities [134]. A comprehensive chemical analysis was conducted to ascertain the primary constituents present in the essential oils derived from mastic (*Pistacia lentiscus* L.). The findings revealed a notable abundance of α-pinene, constituting 70.8% of the composition. Subsequently, β-pinene was identified at a concentration of 5.7%, while myrcene was present at 2.5% [135]. It was reported that mastic gum exhibited a broad spectrum of antimicrobial properties, demonstrating varying degrees of activity against various bacterial and fungal strains, including *Escherichia coli*, *Staphylococcus aureus*, *Bacillus subtilis*, *Helicobacter pylori*, *Streptococcus mutans*, *Microsporum canis*, *Trichophyton mentagrophytes*, and *Trichophyton violaceum* [136]. In a study conducted to assess the industrial applicability of mastic gum essential oils as a natural antimicrobial preservative against spoilage microorganisms in ice cream and juices, the research findings affirmed their suitability for use as potent food preservatives [137].

#### 3.6.19. Nutmeg

Nutmeg is a culinary spice sourced from the seeds of the *Myristica fragrans* Houtt. tree, indigenous to diverse Indonesian islands and additionally cultivated in tropical zones across Southeast Asia. Emanating a distinctively sharp and pleasing aroma, it offers a mildly warm and sweet flavor profile. This spice is used extensively within the food sector. Moreover, it finds utility within traditional medicinal practices, serving as a muscle relaxant, an antiemetic, and a remedy for respiratory ailments, tuberculosis, common colds, and fevers, as well as disorders linked to the nervous and gastrointestinal systems [138]. Nutmeg exhibits a significant phytochemical profile encompassing diverse major classes including anthraquinones, alkaloids, saponins, cardiac glycosides, and flavonoids. The volatile oil of nutmeg is primarily composed of myristicin, a defining compound within a complex amalgamation of terpenes and alkenylbenzene derivatives such as elimicin and safrole. Collectively, these constituents contribute to approximately 80% of the alkenylbenzene derivatives present in nutmeg [139,140]. The acetone extract derived from nutmeg spice has demonstrated noteworthy antibacterial and antifungal efficacy against various microorganisms, including *Staphylococcus aureus* (MTCC 737), *Bacillus subtilis* (MTCC 441), *Pseudomonas putida* (MTCC 1072), and *Pseudomonas aeruginosa* (MTCC 7903), as well as *Aspergillus niger* (MTCC 282), *Aspergillus fumigatus* (MTCC 343), and *Aspergillus flavus* (MTCC 277) [141]. An extended-duration antibacterial evaluation revealed that the encapsulation of nutmeg oil within liposomes had the capacity to prolong and enhance its antibacterial efficacy as a specifically targeted food preservative against *Listeria monocytogenes* in dumplings [142].

#### 3.6.20. Parsley

Parsley, scientifically known as *Petroselinum crispum* (Mill.) Fuss, refers to the aerial consumable components of the plant when used as a spice. Its historical origins can be traced to Sardinia in the Mediterranean region, where it was cultivated as early as 300 BCE. Today, its cultivation spans diverse regions worldwide, and it is valued for its aromatic qualities. In traditional medical practices, parsley has been recommended for addressing gastrointestinal, renal, and lower urinary tract issues. It has been employed in the treatment of conditions such as dyspepsia, cystitis, functional amenorrhea, dysmenorrhea, and myalgia [143]. The principal bioactive constituents identified within parsley are phenolic compounds, predominantly flavonoids, with a notable emphasis on apigenin. Apigenin, a member of the flavonoid family, stands out as a potent agent with significant pharmacological and biological activity [144]. Moreover, the essential oil derived from *Petroselinum crispum* (Mill.) Fuss was subjected to characterization, revealing the predominant presence of specific compounds. Notably, 1,3,8-p-menthatriene constituted 24.2%, β-phellandrene accounted for 22.8%, apiol represented 13.2%, myristicin comprised 12.6%, and terpinolene accounted for 10.3% of the essential oil’s major constituents [145]. Parsley essential oil exhibited notable bacteriostatic effects across the spectrum of tested bacteria, notably against *Listeria monocytogenes*, *Staphylococcus aureus*, and *Salmonella enterica*. These effects were observed at concentrations comparable to or even lower than those of at least one reference control. Furthermore, the essential oil demonstrated pronounced fungistatic activity against all examined fungal strains, primarily *Trichoderma viride* and *Penicillium ochrochloron*. This activity was noticeable at concentrations below those of the comparative ketoconazole control [146].

#### 3.6.21. Rosemary

Rosemary (*Rosmarinus officinalis* L.) is an aromatic herb that is renowned for its culinary and medicinal applications, with an extensive historical utilization spanning diverse cultures. Originally native to the Mediterranean region, its cultivation and utilization have proliferated globally. Appreciated for its fragrant attributes and used as a spice and food seasoning, rosemary has been a staple in traditional medicinal practices, cherished for both its aromatic allure and potential therapeutic contributions. Its traditional medicinal applications encompass the amelioration of renal colic and dysmenorrhea. Additionally, it has found employment in mitigating symptoms associated with respiratory disorders and fostering hair growth; it is also used for anxiety and to enhance alertness [147]. The chemical composition analysis unveiled the substantial presence of rosmarinic acid as the principal compound in rosemary. Alongside this, notable constituents encompass caffeic acid, chlorogenic acid, carnosolic acid, rosmanol, carnosol, various diterpenes, rosmari-diphenoI, rosmariquinonel, glucocolic acid, ursolic acid, and the alkaloid rosmaricine [148]. Considering the emerging applications of natural extracts in the realm of food preservation, the abundance of rosmarinic acid, along with the antioxidant attributes found in rosemary, make it a promising candidate for reducing reliance on synthetic food preservatives. This transition to rosemary-derived preservatives aligns with consumer preferences for natural options and also presents numerous health benefits for consumers [149]. Additionally, research investigations have demonstrated the noteworthy antibacterial efficacy of rosemary essential oils against a range of bacterial strains, including *Bacillus cereus*, *Escherichia coli*, *Staphylococcus aureus*, *Clostridium perfringens*, *Listeria monocytogenes*, *Aeromonas hydrophila*, *Brochothrix thermosphacta*, and *Salmonella choleraesuis* [150].

#### 3.6.22. Saffron

*Crocus sativus* L., also known as saffron, represents a flowering plant of considerable renown, esteemed for its vibrant crimson stigmas. These stigmas are harvested for employment as a culinary spice, as well as for diverse medicinal and cosmetic applications. The historical origins of saffron can be traced back to approximately 1700–1600 BCE, within Greece. Presently, its widespread cultivation is most prominent in Iran and India. Saffron manifests a plethora of medically significant attributes, encompassing antihypertensive, anticonvulsant, antitussive, antigenototoxic, and cytotoxic properties. Additionally, saffron exhibits anxiolytic and aphrodisiac qualities, while also displaying antioxidant, antidepressant, antinociceptive, anti-inflammatory, and relaxant activities [151]. Saffron is characterized by a composition encompassing over 150 volatile, non-volatile, and aroma-contributing constituents. Among these components are crocin, anthocyanin, carotene, lycopene, flavonoids, zeaxanthin, starch, gums, and assorted chemical compounds. Predominantly, the carotenoids, specifically crocin and crocetin, alongside the monoterpene aldehydes of picrocrocin and safranal, stand out as the principal active carotenoid secondary metabolites that are intrinsic to saffron [152]. Given its substantial content of antimicrobial and antioxidant compounds, it is indicated for use as a food preservative [153]. The methanolic and petroleum ether extracts derived from the stigmas of *Crocus sativus* L. (saffron) demonstrated noteworthy bactericidal and fungicidal efficacies against a range of examined microorganisms. Specifically, these microorganisms encompassed *Klebsiella pneumoniae*, *Proteus vulgaris*, *Pseudomonas aeruginosa*, *Escherichia coli*, *Staphylococcus aureus*, *Candida albicans*, *Aspergillus niger*, and *Aspergillus fumigatus* [154].

#### 3.6.23. Thyme

*Thymus vulgaris* L., commonly known as thyme, is native to the Mediterranean region. Its utilization as both a culinary spice and a medicinal agent spans numerous centuries. Thyme boasts an extensive historical legacy in traditional medicine, being harnessed to address a diverse array of ailments. These include its application in the management of respiratory disorders such as whooping cough, bronchitis, and asthma, as well as in the alleviation of urinary tract infections, toothache, and dyspepsia. Thyme is also recognized for its antimicrobial and anti-parasitic attributes, its capacity to stimulate appetite, and its roles as an analgesic and antipyretic agent [155]. Hydro-distilled oils, sourced from both wild and cultivated thyme species, underwent analysis via gas chromatography–mass spectrometry (GC-MS). In total, 24 individual components were successfully identified, collectively comprising a significant proportion ranging from 85% to 98% of the oil’s overall composition. Notably, *Thymus vulgaris*, a cultivated thyme species, demonstrated thymol (35.5% to 44.4%), γ-terpinene (10.5% to 11.9%), carvacrol (4.4% to 16.1%), and p-cymene (8.5% to 16.1%) as the predominant constituents within its oil composition [156]. The essential oil derived from *Thymus vulgaris* exhibited robust antimicrobial characteristics against a spectrum of tested microorganisms. This included notable efficacy against *Pseudomonas aeruginosa* ATCC 27853, *Staphylococcus aureus* ATCC 25923, *Salmonella typhimurium* ATCC 14028, *Klebsiella pneumoniae* ATCC 13882, *Enterococcus faecalis* ATCC 29212, *Escherichia coli* ATCC 25922, *and Candida albicans* ATCC 10231 [157]. Based on the demonstrated efficacy of thyme oil in combating bacterial and fungal growth, its application to cake samples effectively curbed the volatilization of the encapsulated oil and extended the shelf life to a minimum of 30 days, eliminating the need for synthetic preservatives [158].

#### 3.6.24. Turmeric

The rhizomes of *Curcuma longa* L., commonly referred to as turmeric, yield a famous spice that originates from the Indian subcontinent and is presently cultivated extensively in tropical regions across the globe. Curcumin is the major active compound. Empirical investigations have demonstrated that turmeric manifests a range of pharmacological properties, including antimicrobial, anti-parasitic, antispasmodic, antioxidant, anti-inflammatory, and anti-cancer activities. Furthermore, its utilization extends to the management of gastrointestinal disorders [159]. The volatile oil, sourced from the rhizomes of *Curcuma longa* L., was studied using GC-MS analysis of the methanolic extract of curcumin. It revealed the presence of 16 distinct constituents within the oil composition, with 6 specific compounds collectively accounting for 70.0% of the entire oil constituents. The most predominant components were identified as aromatic turmerone (25.3%), α-turmerone (18.3%), and curlone (12.5%). Additional constituents encompassed caryophyllene (2.26%) and eucalyptol (1.60%). Remarkably, the compound found in the most limited quantity was α-phellandrene (0.42%) [160]. Curcumin, the principal constituent of the turmeric rhizome, exhibited notable antimicrobial activity against food spoilage microorganisms and demonstrated strong antioxidant properties. Additionally, its application extended the shelf life of chicken breast fillets to as long as 39 days. Consequently, the utilization of these yellow pigment extracts in food products is advocated for the suppression of lipid oxidation. Moreover, they hold promise as natural food colorants and preservatives, offering a safer alternative to synthetic preservatives that are known for their potential health hazards [161]. Research conducted on the rhizomes of *Curcuma longa* has confirmed its extensive antimicrobial efficacy against a diverse array of microorganisms. Noteworthy examples include, but are not limited to, *Staphylococcus epidermis* ATCC 12228, *Staphylococcus aureus* ATCC 25923, *Klebsiella pneumoniae* ATCC 10031, and *Escherichia coli* ATCC 25922, as well as *Vibrio harveyi*, *Vibrio alginolyticus*, *Vibrio vulnificus*, *Vibrio parahaemolyticus*, *Vibrio cholerae*, *Bacillus subtilis*, *Bacillus cereus*, *Aeromonas hydrophila*, *Streptococcus agalactiae*, *Staphylococcus epidermidis*, *Staphylococcus intermedius*, and *Edwardsiella tarda*, alongside *Candida albicans*, *Cryptococcus neoformans*, and *Fusarium solani* [162].

#### 3.6.25. Vanilla

The vanilla orchid (*Vanilla planifolia* Andrews) has garnered historical acclaim for its applications in both culinary and medicinal domains. Its esteemed position within the culinary realm stems from its extensive tradition as a flavor enhancer. Indigenous to Mexico, the vanilla orchid was originally identified there and was subsequently cultivated within the Mesoamerica region. Scientific investigations have unveiled its potential attributes as an antitumor, anticarcinogenic, and antimicrobial agent, with an effect on anxiety and stress relief along with its capacity to inhibit red blood cell sickling in individuals afflicted with sickle cell disease [163]. Within the array of compounds extracted from the vanilla orchid, the compound vanillin (4-hydroxy-3-methoxybenzaldehyde) plays a pivotal role in conferring the distinct flavor and aroma associated with vanilla. Furthermore, within these extracts, additional flavor constituents, namely, p-hydroxybenzaldehyde, p-hydroxybenzoic acid, and vanillic acid, are also identified within the green vanilla beans [164]. Vanillin represents the principal constituent found in vanilla extract derived from cured vanilla pods. Vanillin finds extensive applications in beverages and food products, and it enjoys a recognized safety status, having been approved by both the Food and Drug Administration (FDA) and the Flavor and Extract Manufacturer Association (FEMA) for use as a food additive. Given its inherent antimicrobial and antioxidant properties, vanillin holds great potential for utilization as a food preservative [165]. Numerous research investigations have indicated the potential antimicrobial attributes associated with specific constituents that are present in vanilla, including vanillin and other phenolic compounds. These properties have been observed against a range of microorganisms, encompassing, but not limited to, *Staphylococcus saprophyticus*, *Staphylococcus aureus*, *Staphylococcus epidermidis*, *Streptococcus pyogenes*, *Enterococcus faecalis*, *Enterobacter cloacae*, *Enterobacter hormaechei*, *Salmonella typhimurium*, *Klebsiella pneumoniae*, *Escherichia coli*, and *Pseudomonas aeruginosa* [166].

**Table 1:** Prominent spices exhibiting remarkable antimicrobial activities, proposed as natural preservatives for the food industry.

No.	Spice	Scientific Name	Botanical Family	Major Bioactive Compound	Chemical Structure	Antimicrobial Activity	Ref.
1	Allspice	*Pimenta dioica* (L.) Merr.	Myrtaceae	Eugenol	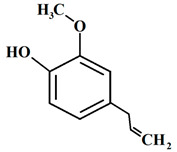	*Listeria monocytogenes* CECT 933, *Vibrio vulnificus* CECT 529, *Salmonella enterica* CECT 443, *Shigella flexeneri* CECT 4804, *Escherichia coli* ATCC 35218, *Staphylococcus aureus* ATCC 6538, and *Aspergillus flavus*.	[58,59]
2	Anise	*Pimpinella anisum* L.	Apiaceae	Anethole	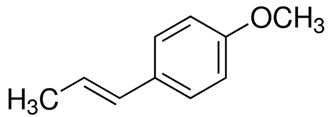	*Escherichia coli* ATCC 25922, *Staphylococcus aureus* ATCC 25923, *Pseudomonas aeruginosa* ATCC 27853, *Streptococcus pyogenes* ATCC 19615, and *Candida albicans* ATCC 10231.	[61,62]
3	Basil	*Ocimum basilicum* L.	Lamiaceae	Methyl cinnamate	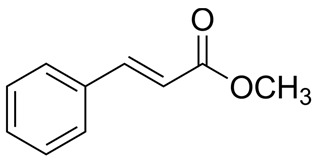	*Staphylococcus epidermidis* MTCC 435, *Streptococcus mutans* MTCC 890, *Escherichia coli* MTCC 723, *Candida kefyr* ATCC 204093, and *Candida albicans* ATCC 14053.	[66,67]
4	Bell pepper	*Capsicum annuum* L.	Solanaceae	Capsaicinoids	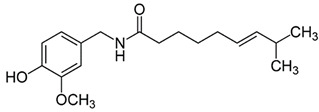	*Listeria monocytogenes*, *Salmonella typhimurium*, *Staphylococcus aureus*, *Escherichia coli*, *Bacillus subtilis*, *Proteus mirabilis*, *Lactobacillus plantarum*, *Lactobacillus acidophilus*, and *Cochliobolus* spp.	[70,71]
5	Black Pepper	*Piper nigrum* L.	Piperaceae	Piperine	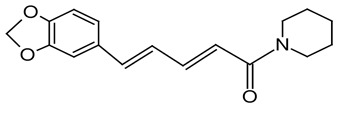	*Klebsiella pneumonia* ATCC 27853, *Escherichia coli* ATCC 25922, *Salmonella enterica* ATCC 43972, *Staphylococcus aureus* ATCC 25923, *Enterococcus faecalis* ATCC 29122, *Staphylococcus epidermidis* ATCC 14990, *Bacillus subtilis* ATCC 6633, and *Aspergillus flavus* CGMCC 3.06434.	[75,76]
6	Black seeds	*Nigella sativa* L.	Ranunculaceae	Thymoquinone	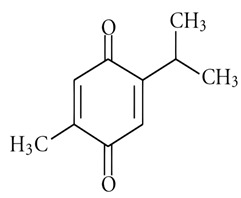	*Escherichia coli*, *Klebsiella pneumoniae*, *Staphylococcus aurous*, *Enterobacter aerogenes*, *Fusarium Solani*, *Candida albicans* AUMC 1299, *Aspergillus flavus* AUMC 1276, *Fusarium oxysporum* AUMC 215, *Scopulariopsis brevicaulis* AUMC 1653, *Geotrichum candidum* AUMC 226, and *Trichophyton rubrum* AUMC 1804.	[81,82]
7	Caraway	*Carum carvi* L.	Apiaceae	Carvone	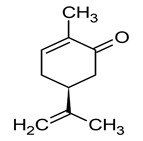	*Escherichia coli*, *Pseudomonas aeruginosa*, *Staphylococcus aureus*, *Bacillus cereus*, and *Aspergillus flavus*.	[86,87,88]
8	Cardamom	*Elettaria cardamomum* (L.) Maton	Zingiberaceae	Cardamonin	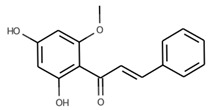	*Bacillus subtilis*, *Staphylococcus aureus*, *Pseudomonas aeruginosa* and *methicillin-resistant Staphylococcus aureus* (ATCC 43300), *Salmonella typhimurium* (ATCC 14023), and *Escherichia coli* (ATCC 25922).	[90,92,93]
9	Cinnamon	*Cinnamomum verum* J.Presl	Lauraceae	Cinnamaldehyde	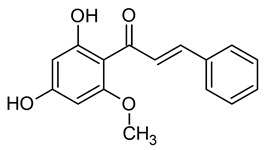	*Micrococcus luteus*, *Bacillus subtilis*, *Bacillus cereus*, *Klebsiella aerogenes*, *Escherichia coli*, *Salmonella enterica*, *Penicillium expansum*, *Candida albicans*, and *Candida tropicalis*.	[95,96]
10	Clove	*Syzygium aromaticum* (L.) Merr. & L.M.Perry	Myrtaceae	Eugenol	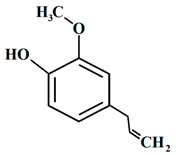	*Staphylococcus aureus*, *Listeria innocua*, *Pseudomonas aeruginosa*. *Serratia marcescens*, *Bacillus subtilis*, *Penicillium commune*, *Penicillium expansum*, *Penicillium glabrum*, and *Penicillium chrysogenum*	[99,100,101]
11	Coriander	*Coriandrum sativum* L.	Apiaceae	Linalool	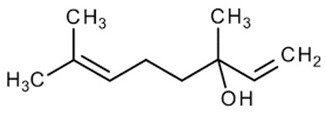	*Bacillus subtilis*, *Stenotrophomonas Penicillium expansum*, *Streptococcus pyogenes*, *Listeria monocytogenes*, *Enterobacter aerogenes*, *Salmonella typhimurium*, and *Shigella dysenteriae*.	[104,105,106]
12	Cumin	*Cuminum cyminum* L.	Apiaceae	Cuminaldehyde	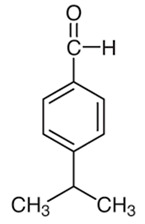	*Staphylococcus aureus*, *Bacillus cereus*, *Escherichia coli*, *Salmonella typhi*, *Botrytis cinerea*, *Penicillium expansum*, and *Aspergillus niger*.	[108,109,110]
13	Dill	*Anethum graveolens* L.	Apiaceae	Carvone	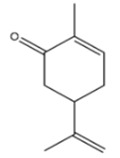	*Escherichia coli*, *Staphylococcus aureus*, *Yersinia enterocolitica*, *Geotrichum candidum*, *Salmonella typhimurium*, *Rhodotorula glutinis*, *Saccharomyces cerevisiae*, and *Candida albicans*.	[112,113]
14	Fennel	*Foeniculum vulgare* Mill.	Apiaceae	Anethole	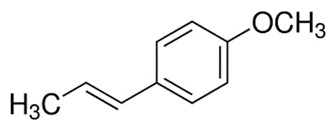	*Staphylococcus albus*, *Bacillus subtilis*, *Salmonella typhimurium*, *Shigella dysenteriae*, *Escherichia coli*, *Bacillus cereus*, *Staphylococcus aureus*, *Candida albicans*, and *Aspergillus flavus*.	[117,118,119]
15	Fenugreek	*Trigonella foenum-graecum* L.	Fabaceae	Sotolone	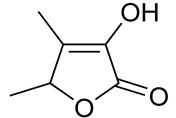	*Staphylococcus aureus* ATCC 25923, *Escherichia coli* ATCC 25922, *Bacillus subtilis* NCTC 8236, *Pseudomonas aeruginosa* ATCC 27853, *Aspergillus niger* ATCC 9763, and *Candida albicans* ATCC 7596.	[121,122]
16	Garlic	*Allium sativum* L.	Amaryllidaceae	Allicin	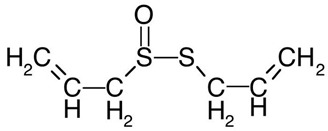	*Staphylococcus saprophyticus*, *Staphylococcus aureus*, *Staphylococcus epidermidis*, *Bacillus cereus*, *Streptococcus pneumoniae*, *Shigella flexneri*, *Proteus vulgaris*, *Klebsiella pneumoniae*, *Escherichia coli*, *Aspergillus versicolor*, *Penicillium expansum*, *Penicillium citrinum*, and *Candida albicans*.	[125,127,128]
17	Ginger	*Zingiber officinale* Roscoe	Zingiberaceae	Gingerol	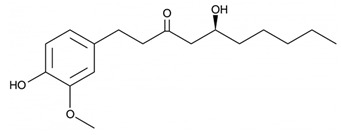	*Staphylococcus epidermidis*, *Staphylococcus aureus*, *Enterococcus faecalis*, *Streptococcus faecalis*, *Bacillus subtilis*, *Bacillus megaterium*, *Bacillus cereus*, *Escherichia coli*, *Klebsiella pneumoniae*, *Salmonella typhimurium*, *Salmonella typhi*, *Pseudomonas aeruginosa*, *Proteus* spp., *Aspergillus niger*, *Aspergillus flavus*, *Penicillium expansum*, *Alternaria alternata*, *Fusarium oxysporum*, *Mucor hemalis*, *Penicillium notatum*, *Candida albicans*, and *Fusarium oxysporum*.	[130,132,133]
18	Mastic	*Pistacia lentiscus* L.	Anacardiaceae	α-pinene	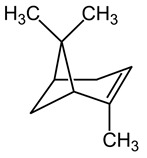	*Escherichia coli*, *Staphylococcus aureus*, *Bacillus subtilis*, *Helicobacter pylori*, *Streptococcus mutans*, *Microsporum canis*, *Trichophyton mentagrophytes*, and *Trichophyton violaceum*.	[135,136]
19	Nutmeg	*Myristica fragrans* Houtt.	Myristicaceae	myristicin	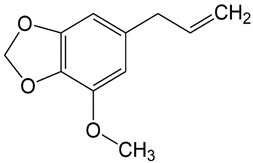	*Staphylococcus aureus* MTCC 737, *Bacillus subtilis* MTCC 441, *Pseudomonas putida* MTCC 1072, *Pseudomonas aeruginosa* MTCC 7903, *Listeria monocytogenes*, *Aspergillus flavus* MTCC 277, *Aspergillus niger* MTCC 282, and *Aspergillus fumigatus* MTCC 343.	[140,141,142]
20	Parsley	*Petroselinum crispum* (Mill.) Fuss	Apiaceae	Apigenin	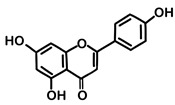	*Salmonella enterica*, *Staphylococcus aureus*, *Listeria monocytogenes*, *Penicillium ochrochloron*, and *Trichoderma viride*	[144,146]
21	Rosemary	*Rosmarinus officinalis* L.	Lamiaceae	Rosmarinic acid	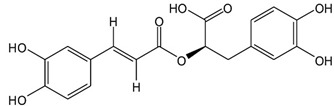	*Bacillus cereus*, *Staphylococcus aureus*, *Salmonella choleraesuis*, *Clostridium perfringens*, *Aeromonas hydrophila*, *Escherichia coli*, *Listeria monocytogenes*, and *Brochothrix thermosphacta*.	[149,150]
22	Saffron	*Crocus sativus* L.	Iridaceae	Crocin	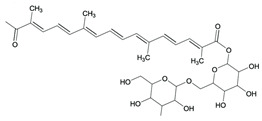	*Klebsiella pneumoniae*, *Pseudomonas aeruginosa*, *Proteus vulgaris*, *Staphylococcus aureus*, *Escherichia coli*, *Candida albicans*, *Aspergillus fumigatus*, and *Aspergillus niger*.	[152,154]
23	Thyme	*Thymus vulgaris* L.	Lamiaceae	Thymol	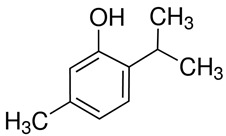	*Pseudomonas aeruginosa* ATCC 27853, *Staphylococcus aureus* ATCC 25923, *Salmonella typhimurium* ATCC 14028, *Klebsiella pneumoniae* ATCC 13882, *Enterococcus faecalis* ATCC 29212, *Escherichia coli* ATCC 25922, and *Candida albicans* ATCC 10231.	[156,157]
24	Turmeric	*Curcuma longa* L.	Zingiberaceae	Curcumin	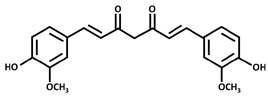	*Staphylococcus aureus* ATCC 25923, *Staphylococcus epidermis* ATCC 12228, *Escherichia coli* ATCC 25922, *Klebsiella pneumoniae* ATCC 10031, *Vibrio harveyi*, *Vibrio cholerae*, *Bacillus subtilis*, *Bacillus cereus*, *Aeromonas hydrophila*, *Staphylococcus intermedius*, *Edwardsiella tarda*, *Streptococcus agalactiae*, *Cryptococcus neoformans*, *Candida albicans*, and *Fusarium solani*.	[161,162]
25	Vanilla	*Vanilla planifolia* Andrews	Orchidaceae	Vanillin	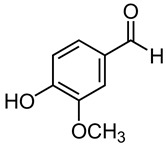	*Staphylococcus epidermidis*, *Staphylococcus saprophyticus*, *Staphylococcus aureus*, *Streptococcus pyogenes*, *Enterococcus faecalis*, *Enterobacter hormaechei*, *Enterobacter cloacae*, *Klebsiella pneumoniae*, *Salmonella typhimurium*, *Escherichia coli*, and *Pseudomonas aeruginosa*.	[165,166]

## 4. Future Prospective

In recent years, the exploration of spice-derived compounds for food preservation has garnered significant attention from researchers and food industry professionals alike. The next phase of research should focus on developing precise and controlled formulations of spice-derived compounds. To ensure consistent antimicrobial efficacy in various food matrices, this entails identifying the ideal concentration against spoilage microorganisms that is unaffected by encapsulation techniques or storage systems. Delving deeper into the mechanisms behind the antimicrobial activity of spice-derived compounds is crucial. Advanced techniques, such as omics approaches and molecular modeling, can provide insights into the specific interactions between these compounds and microbial targets. Future research should address the safety aspects of spice-derived compounds. This includes evaluating potential allergy reactions, toxicity, and any unintended consequences of long-term exposure to these compounds in preserved foods. Beyond traditional food preservation, researchers can explore novel applications for spice-derived compounds, such as their use in food packaging, antimicrobial coatings, and edible films to extend shelf life and enhance food safety. Investigating synergistic combinations of spice-derived compounds with other natural antimicrobials or conventional preservatives could amplify their effectiveness while reducing the risk of microbial resistance development. As spice-derived compounds move closer to practical food preservation applications, it becomes essential to establish clear regulations. Understanding consumer perceptions of foods preserved with spice-derived compounds and their willingness to embrace such products is pivotal for successful market penetration and commercial viability. However, guidelines and standards to ensure consumer safety and promote industry adoption are essential. Exploring the sustainability aspects of spice-derived preservation methods, including the environmental impact and resource utilization, aligns with global sustainability goals and the growing demand for eco-friendly food preservation solutions. Finally, future research should encourage collaborations between food scientists, microbiologists, chemists, and experts from diverse fields to foster a holistic approach to spice-derived compound applications in food preservation.

## 5. Conclusions

The use of spices is prehistoric. In vitro and in vivo studies have examined their role in food safety and preservation, in addition to their primary use as flavoring and coloring compounds. Spices can prevent and treat cancer, aging, and metabolic, neurological, cardiovascular, and inflammatory problems. Spices contain antibacterial and antifungal chemicals that can kill spoilage microorganisms and fight human infections. Numerous spices harbor noteworthy antimicrobial agents, presenting a potential source of inhibitory substances against food spoilage and foodborne pathogens. Despite this, limited information exists on the preservative and antimicrobial roles of spices in preventing microbial activity in food. The majority of these studies have been conducted in vitro, with only a few attempts being made to assess their antimicrobial potential in real food systems as alternatives to artificial preservatives. Furthermore, the efficacy of spices in inhibiting food spoilage microorganisms during their culinary use remains unexplored. In this regard, the present review identifies 25 spice types with reported antimicrobial activity. Consequently, research centers specializing in the food industry play a crucial role in investigating the compounds within these spices and determining the most suitable candidates for application as natural and safe preservatives. Such efforts would stimulate a heightened demand for products preserved with natural compounds, aligning with the rising preference for natural food additives and preservatives among consumers. A thorough understanding of the precise mechanisms underlying the action of phytochemicals is essential to developing novel antibacterial agents. Moreover, it is crucial to consider potential interactions with food ingredients that could impact the antimicrobial effectiveness of spice-derived agents. As consumer demand for natural food additives and preservatives rises, exploring the antimicrobial properties of spices presents a promising alternative. However, the objective should entail a comprehensive approach involving a combination of processes and the judicious use of low concentrations of preservatives, either individually or in conjunction with natural antimicrobials, to ensure the production of safe and high-quality food products.

## Data Availability

Not applicable.
